# Changes of thrombelastography in patients undergoing elective primary total knee and total hip replacement with low molecular heparin prophylaxis

**DOI:** 10.1186/s13018-014-0052-0

**Published:** 2014-07-08

**Authors:** Yi Yang, Zhenjun Yao, Wenda Dai, Peng Shi, Lei luo, Chi Zhang

**Affiliations:** 1 Department of Orthopaedic, Zhongshan Hospital of Fudan University, Shanghai 200032, China; 2 Department of Clinical Epidemiology, Children’s Hospital of Fudan University, Shanghai 201102, China; 3 Department of Laboratory, Zhongshan Hospital of Fudan University, Shanghai 200032, China

**Keywords:** Thromboprophylaxis, Low molecular weight heparin, Arthroplasty, Thrombelastography, Hypercoagulability

## Abstract

**Background:**

There has been no effective method to monitor the changes of blood coagulation after thromboprophylaxis for elective arthroplasty patients. The objective of this study is to assess the coagulation status of patients undergoing arthroplasty with thromboelastograph (TEG).

**Methods:**

Ninety patients undergoing primary elective unilateral arthroplasty were investigated. Thromboprophylaxis continued for at least 10 days. TEG was performed on the day before the operation and on postoperative days 1, 4, and 9.

**Results:**

The total hip and total knee groups showed significant changes in the distribution of different hypercoagulable states on days 1–4 and on days 4–9. On day 9 after operation, 34 out of 90 (37.8%) of the total hip and total knee patients were found with hypercoagulable state. Of these 34 patients with hypercoagulable state, 26 (76.5%) demonstrated platelet or mixed hypercoagulability.

**Conclusions:**

Thrombelastography was an effective way to identify hypercoagulability in patients undergoing elective primary total knee and total hip replacement. Platelet may play an important role in the progress of blood hypercoagulability.

## Background

Venous thromboembolism (VTE), including pulmonary embolism (PE) and deep vein thrombosis (DVT), is a severe complication in major orthopedic surgery. The incidence of venous thromboembolism following total joint replacement (TJR) has diminished over the last three decades [[1]]. The routine use of anticoagulants after total knee and total hip replacement is strongly recommended by, at present, the guidelines by the American Association of Chest Physicians (ACCP) [[2]]. However, with the routine use of thromboprophylaxis, some patients still develop DVT of the lower extremity and PE, while a minority of them may be at risk for bleeding complications [[2]–[4]]. This suggests that it is important to accurately monitor the changes of coagulation after anticoagulation during perioperative period.

Thromboelastograph (TEG) is a point-of-care test for evaluation of hemostasis, which has been widely used in the field of liver transplantation and coronary bypass surgery as an intraoperative hemostatic monitoring device [[5]– [7]]. By measuring the dynamic process of blood coagulation, with defined parameters reflecting integrity of specific hemostatic components, this device can differentiate hypercoagulable state into different types—platelet, enzymatic, and mixed, according to the manufacturer [[8]]. But monitoring blood coagulation with thromboelastograph had not gained popularity in the field of orthopedics [[9],[10]].

The objective of this study is to assess the coagulation status of patients undergoing arthroplasty with TEG.

### Patients and methods

This study was conducted prospectively and approved by the hospital’s ethics committee. Ninety patients (mean age 64 ± 2 years) undergoing primary elective unilateral total knee or total hip replacement were investigated, with 48 patients for knee and 42 for hip. Informed consent was obtained from each patient. The coagulation functions of all patients were normal before operation. None of the patients has a history of heparin-induced thrombocytopenia (HIT) or kidney insufficiency (CrCl < 30 ml/min).

All total knee and total hip replacements were performed by the same group of surgeons, Genesis II knee system of Smith & Nephew was used in the knee replacements, Synergy hip system of Smith & Nephew, and Summit hip system of Johnson & Johnson were used in the hip replacements. All operations were performed under general anesthesia. No transfusion of more than 2 units of RBC within 6 h in perioperative period was done.

Fraxiparine, a type of low weight molecular heparin (nadroparin calcium, 9,500 anti-Xa IU/mL), was used as routine thromboprophylaxis after joint replacement. Single daily doses of Fraxiparine were adjusted according to the patient’s body weight as follows: 38 anti-Xa IU/kg administered 12 h after surgery, 38 anti-Xa IU/kg re-administered on a daily basis, up to and including postoperative day 3, and 57 anti-Xa IU/kg administered since postoperative day 4. Thromboprophylaxis continued for at least 10 days.

TEG was performed on the day before the operation; 0.36 mL of whole blood was pipetted into a disposable plastic cup within 4 min of blood sampling. A stationary pin attached to a wire which can monitor movements is immersed into the sample. The cup oscillates back and forth six times per minute. A computerized thromboelastograph coagulation analyzer (TEG model 5000; Haemoscope Corporation, Niles, IL, USA) was used in this study. After the subcutaneous injection of nadroparin sodium, TEG was performed at 4 h on postoperative days 1, 4, and 9.

TEG values include R (reaction time; time to initial thrombus formation), K (rate of thrombus formation), MA (maximum amplitude; thrombus strength), α-angle (rate of thrombus formation), and CI (coagulation index). CI is a computer-calculated linear combination of the R, K, MA, and α-angle values, and reflects overall coagulation status.

TEG-hypercoagulability was classified into three types: (1) enzymatic hypercoagulability, CI > 3, R < = 5 min, MA < = 70 mm; (2) platelet hypercoagulability, CI > 3, R > 5 min, MA > 70 mm; (3) mixed hypercoagulability: CI > 3, R < = 5 min, MA > 70 mm, according to the manufacturer.

### Statistical analysis

Data were presented as means ± standard deviation for continuous variables with normal distribution and *n* (%) for category variables. Student *t* test was used to compare the means of continuous variable with normal distribution, and Chi-square test was used to compare the proportion of category variable between the total hip group and total knee group. Linear mixed model and estimating equations (GEE) approach was used to analyze the repeated measurement of continuous data and categorical data, respectively. The proportion of different kinds of hypercoagulability at four time points was compared using Fisher’s exact test.

All statistical analysis was conducted using SAS 9.1.3 (SAS Institute Inc., Cary, NC, USA). *P* < 0.05 was regarded as statistically significant.

## Results

The characteristics of patients before operation were shown in Table 1. The mean age of patients was 66.7 and 71.5 years for total hip group and total knee group, respectively (*P* < 0.0166). The proportion of sex and means of height, weight, and BMI between these two groups were not statistically significant (*P* > 0.05).

**Table 1:** The baseline characteristics of patients before operation

	**Total hip group**	**Total knee group**	**Total**	***P*** **value**
	***n*** **= 42**	***n*** **= 48**	***n*** **= 90**	
Age (years)				
Mean ± SD	66.7 ± 9.7	71.5 ± 7.6	69.2 ± 8.9	0.0104
Min, max	43, 81	54, 82	43, 82	
Sex, *n* (%)				
Male	13 (31.0)	7 (14.6)	20 (22.2)	0.0624
Female	29 (69.0)	41 (85.4)	70 (77.8)	
Height (cm)				
Mean ± SD	163.2 ± 7.7	161.2 ± 7.7	162.2 ± 7.7	0.2427
Min, max	150, 183	150, 186	150, 186	
Weight (kg)				
Mean ± SD	63.3 ± 9.9	65.1 ± 10.3	64.3 ± 10.1	0.4208
Min, max	46, 82	43, 89	43, 89	
BMI (kg/m2)				
Mean ± SD	23.8 ± 3.2	24.4 ± 3.4	24.6 ± 3.5	0.1074
Min, max	19.1, 32.5	18.9, 35.8	18.9, 35.8	

*P* value was calculated by Chi-square test for category variable and one-way ANOVA for continuous variable.

1. Change of TEG between the two patients groups. The differences in values of R, K, MA, α-angle, and coagulation index (CI) between the two patient groups were not statistically significant before operation and on days 1, 4, and 9 after operation (Table 2). There were no significant differences in the response categories (normal, enzymatic, platelet, and mixed hypercoagulability) between the total hip group and total knee group (*P* = 0.0893).

**Table 2:** Effect of low molecular heparin prophylaxis on the thrombelastography between the two patient groups at different time points

	**Total hip group**	**Total knee group**	**Total**	***P*** **value**
	***n*** **= 42**	***n*** **= 48**	***n*** **= 90**	
*R* (mm)				
Before	5.68 ± 0.92	5.86 ± 1.03	5.78 ± 0.98	0.4830
Day1	5.30 ± 1.04	5.63 ± 1.32	5.47 ± 1.20	0.1586
Day4	5.25 ± 0.98	5.56 ± 1.04	5.41 ± 1.02	0.1886
Day9	5.32 ± 1.12	5.68 ± 1.19	5.52 ± 1.17	0.1255
*K* (mm)				
Before	1.90 ± 0.78	1.80 ± 0.39	1.85 ± 0.59	0.3866
Day1	1.61 ± 0.41	1.79 ± 0.54	1.71 ± 0.49	0.0847
Day4	1.35 ± 0.36	1.40 ± 0.34	1.38 ± 0.35	0.6934
Day9	1.30 ± 0.65	1.25 ± 0.35	1.28 ± 0.50	0.6763
MA (mm)				
Before	61.15 ± 6.00	62.00 ± 5.34	61.63 ± 5.61	0.5607
Day1	63.20 ± 5.29	61.41 ± 4.50	62.27 ± 4.95	0.1658
Day4	66.81 ± 7.31	66.33 ± 6.23	66.55 ± 6.73	0.7103
Day9	69.35 ± 6.48	69.14 ± 6.84	69.24 ± 6.64	0.8783
α-angle				
Before	67.76 ± 5.57	67.79 ± 5.17	67.78 ± 5.31	0.9784
Day1	69.13 ± 5.72	67.63 ± 6.64	68.34 ± 6.23	0.1844
Day4	72.15 ± 5.62	72.23 ± 3.85	72.19 ± 4.75	0.9450
Day9	74.06 ± 4.89	73.66 ± 4.65	73.84 ± 4.74	0.7273
CI				
Before	0.42 ± 1.76	0.46 ± 1.34	0.44 ± 1.53	0.9251
Day1	1.12 ± 1.58	0.45 ± 1.89	0.77 ± 1.77	0.0455
Day4	1.94 ± 1.54	1.67 ± 1.26	1.80 ± 1.40	0.4236
Day9	2.23 ± 1.73	2.09 ± 1.52	2.15 ± 1.61	0.6695

Data are reported as mean ± SD.

**Figure 1:**
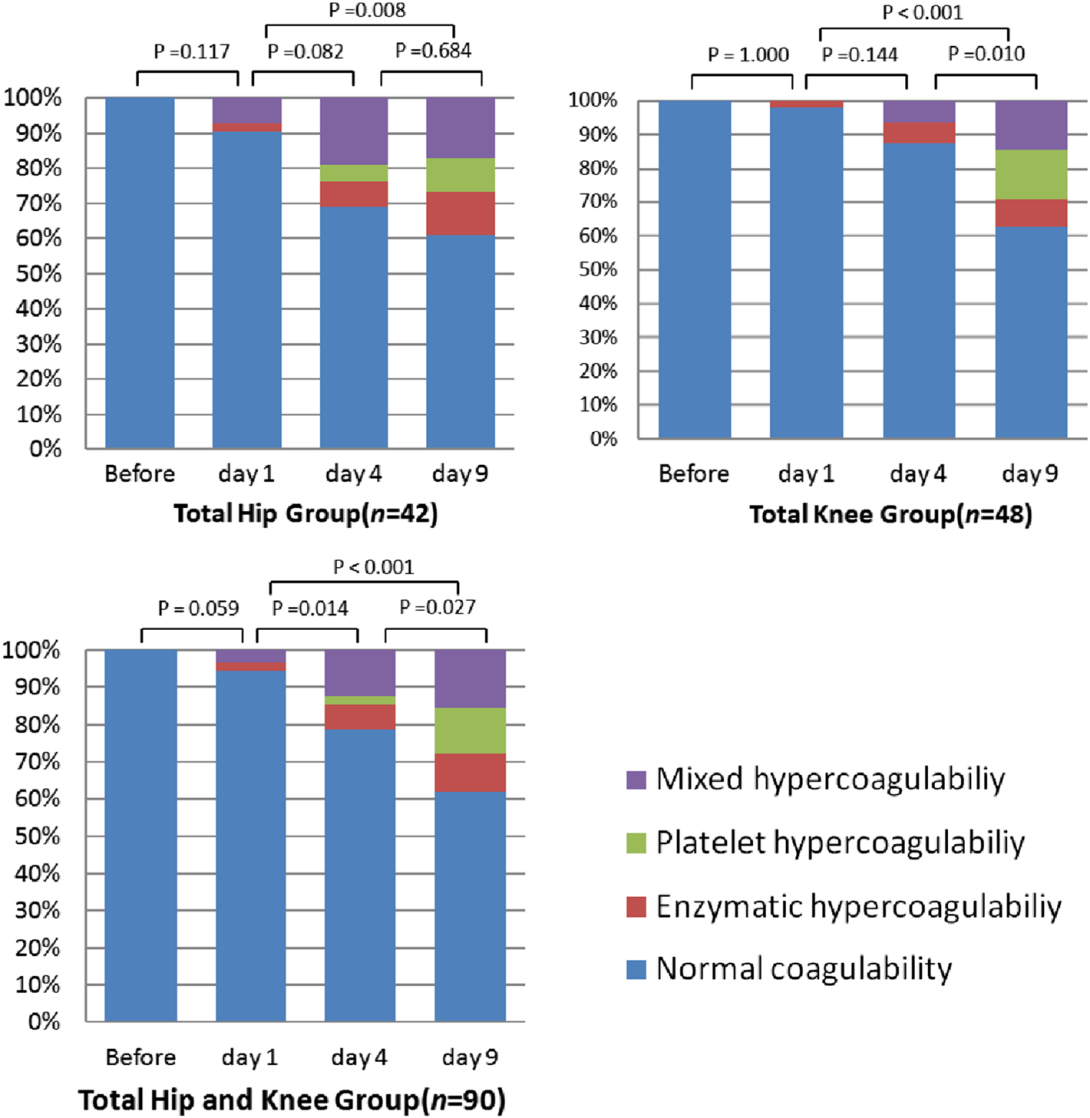
Proportion of different status of hypercoagulability at different time points.

2. Changes of hypercoagulable states. There were no significant changes in the R, K, MA, α-angle, CI before operation, and day 1 after operation. However, significant change in the K, MA, α-angle, and CI was observed on days 1–4 after operation. The changes in the MA and α-angle were significant on days 4–9 after operation (Figure 1).

**Figure 2:**
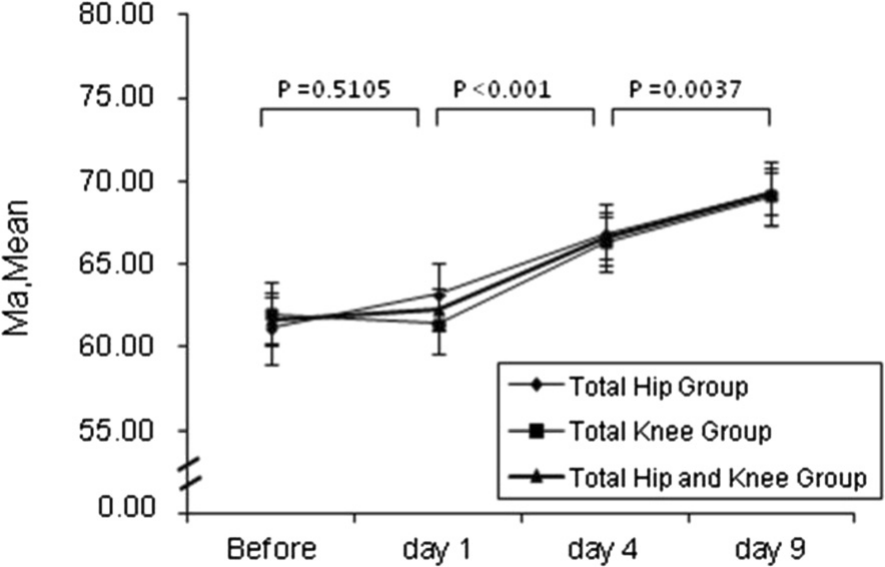
**Trends of MA after operation between three groups.** I bars indicate the 95% confidence intervals. *P* value is the comparison between different time points of the total hip and knee group.

The distribution of different hypercoagulable states before and after operation in the total hip group and total knee group were shown in Figure 2. As there were no significant differences in the response categories between the two patient groups, the pooled total hip and total knee groups showed significant changes in the distribution of different hypercoagulable states on days 1–4 and on days 4–9 (Figure 2). On day 9 after operation, 34 out of 90 (37.8%) of the total hip and total knee patients were found with hypercoagulable state. Of these 34 patients with hypercoagulable state, 26 (76.5%) demonstrated platelet or mixed hypercoagulability.

## Discussion

In patients undergoing elective total hip and total knee arthroplasty, multiple factors disrupt the regulatory mechanisms of hemostasis, such as endothelial injury, stasis, and platelet activation [[10]–[12]]. These factors may result in a hypercoagulable state. As we know, hypercoagulability has been implicated in the pathogenesis of VTE events [[9],[10],[12],[13]]. So both the AAOS and the ACCP9 recommended to prevent VTE after elective joint replacement [[2],[3]]. But even with appropriate thromboprophylaxis, a certain proportion of patients still showed hypercoagulable tendency. Patel et al. concluded in multicenter study that most VTE’s occurred due to prophylaxis failure rather than failure to provide prophylaxis [[14]].

Until now, there has been no effective method to ensure adequate thromboprophylaxis with careful monitoring. Because the curve of TEG reflects the different phases of the clotting process and enables a qualitative evaluation of the individual steps involved, recent studies suggested that TEG could be used to identify hypercoagulable state in a variety of clinical settings, and have revealed an association between hypercoagulability measured by thrombelastography and postoperative/postinterventional thromboembolic complications [[5],[8]–[10],[13]]. Park et al. reported that thromboelastography could be taken as a better indicator of postinjury hypercoagulable state than prothrombin time or activated partial thromboplastin time [[15]]. It was also suggested that evoked hypercoagulability in the early postoperative period was important for predicting TE complications [[16]]. An observational study in patients undergoing major noncardiac surgery found that 8 out of 95 (8.4%) of TEG-hypercoagulable patients had a postoperative thromboembolic complication, while only 2 out of 145 (1.4%) of such patients experienced thromboembolic episodes (*P* = 0.016) [[17]].

According to the classification of the TEG standard, hypercoagulable patients fall into different types, including enzymatic, platelet, and mixed hypercoagulability. Our study found 38.1% total hip patients and 37.5% total knee patients with hypercoagulable states on day 9 postoperation. For most of these patients, their hypercoagulable states could be classified into mixed hypercoagulability or platelet hypercoagulability, which means MA values are greater than 70 mm. MA is dependent on platelet concentration, platelet function, and platelet-fibri interaction [[6]]. These findings indicated a marked increase in the platelet factors related to the hypercoagulability while thromboprophylaxis was performed with low weight molecular heparin. Traditionally, endothelial injury and platelet activation are known to be triggers for arterial thromboemboli. Arterial and venous thromboses have been viewed as distinct conditions, with differences in risk factors, pathology, and treatment [[18]]. But several lines of evidence suggested that activation of platelets did indeed contribute to the development and propagation of venous thrombi [[19]]. Chirinos et al. reported that activation of the endothelium, platelets, and leukocytes occurred in patients with VTE, and the formation of platelet-leukocyte conjugates regulated leukocyte activation and participated in linking thrombosis with inflammation *in vivo* [[20]]. In a rabbit model of VTE, Takahashi et al. [[21]] demonstrated that an antibody against von Willebrand factor (vWF) (AJW200), which inhibited interactions between A1 domain and platelet GPIb, significantly reduced venous thrombus formation and pulmonary thromboembolism. These results suggest that VWF A1-platelet GPIb interaction played a significant role in venous thrombus formation. Moreover, inhibition of P-selectin, a signaling molecule exposed on the surface of an activated platelet, which initiates inflammatory signaling pathways in underlying endothelium and recruits monocytes, resulted in impaired thrombus formation both in an experimental model of venous thrombosis and *in vivo* [[22]]. Thus, as Gonzalez et al. [[8]] suggested, solely targeting final thrombin production by utilizing UH or LMWH may not provide enough protection against platelet activation, hence a hypercoagulable state may persist.

Bozic et al. [[23]] found that patients who received aspirin VTE prophylaxis had lower odds for thromboembolism compared with warfarin patients but had similar odds compared with those with injectable VTE prophylaxis. ACCP9 recommended aspirin monotherapy as a method for thromboprophylaxis after the joint arthroplasty. The increase in MA values in our study confirmed that platelet played an increasingly important role in the progress of blood hypercoagulability.

It was also found in the current study that some patients appeared to be enzymatic hypercoagulable after anticoagulation. The simplest explanation for this would be that the recommended dose of low molecular weight heparin was insufficient. Since the exact effective dose of LMWH for prophylaxis varies from patient to patient, it is necessary to monitor the effects of the administered LMWH to ensure that each individual patient is adequately anticoagulated without bleeding tendency. TEG is a useful technique for rapid global assessment of hemostatic function while the patient is receiving thromboprophylaxis after joint arthroplasty. Since ACCP9 recommend the use of pharmacologic thromboprophylaxis for a minimum of 10 to 14 days (Grade 1B), we believed that TEG data on postoperation day 9 would be a potential measurement to determine whether patients need extended prophylaxis or not. However, lack of TEG data on day 35 was one limitation of our study.

In conclusion, our results indicate that thrombelastography was an effective way to identify hypo- and hypercoagulability in patients undergoing elective primary total knee and total hip replacement. Under recommended dose of LMWH, over 1/3 of patients were in hypercoagulability on postoperative day 9. Furthermore, we found that platelet may play an important role in the progress of blood hypercoagulability.

## Competing interests

The authors declare that they have no competing interests.

## Authors’ contributions

The design of the study and preparation of the manuscript were done by YY, CZ, and ZY. WD assisted in the manuscript preparation. PS performed the statistical analysis. LL assisted in the study processes and data collections. All authors read and approved the final manuscript.

## References

[B1] Freedman KB Brookenthal KR Fitzgerald RH Jr Williams S Lonner JH A meta-analysis of thromboembolic prophylaxis following elective total hip arthroplasty J Bone Joint Surg Am 2000 82-A 7 929 938 10901307 10.2106/00004623-200007000-00004

[B2] Prevention of VTE in orthopedic surgery patients: antithrombotic therapy and prevention of thrombosis, 9th ed: American College of Chest Physicians Evidence-Based Clinical Practice Guidelines Chest 2012 141 2 Suppl e278S e325S 22315265 10.1378/chest.11-2404 PMC3278063

[B3] Mont MA Jacobs JJ Boggio LN Bozic KJ Della Valle CJ Goodman SB Lewis CG Yates AJ Jr Watters WC 3rd Turkelson CM Wies JL Donnelly P Patel N Sluka P Preventing venous thromboembolic disease in patients undergoing elective hip and knee arthroplasty J Am Acad Orthop Surg 2011 19 12 768 776 22134209 10.5435/00124635-201112000-00007

[B4] Bloomfield MR Patterson RW Froimson MI Complications of anticoagulation for thromboembolism in early postoperative total joint arthroplasty Am J Orthop (Belle Mead NJ) 2011 40 8 E148 E151 22016874

[B5] Rafiq S Johansson PI Ostrowski SR Stissing T Steinbrüchel DA Hypercoagulability in patients undergoing coronary artery bypass grafting: prevalence, patient characteristics and postoperative outcome Eur J Cardiothorac Surg 2012 41 3 550 555 10.1093/ejcts/ezr001 22011771

[B6] Reikvam H Steien E Hauge B Liseth K Hagen KG Størkson R Hervig T Thrombelastography Transfus Apher Sci 2009 40 2 119 123 10.1016/j.transci.2009.01.019 19249246

[B7] Kouerinis IA Kourtesis A El-Ali M Sergentanis T Plagou A Argiriou M Theakos N Giannakopoulou A Heparin induced thrombocytopenia diagnosis in cardiac surgery: is there a role for thromboelastography? Interact Cardiovasc Thorac Surg 2008 7 4 560 563 10.1510/icvts.2007.161679 18056152

[B8] Gonzalez E Kashuk JL Moore EE Silliman CC Differentiation of enzymatic from platelet hypercoagulability using the novel thrombelastography parameter delta (△) J Surg Res 2010 163 1 96 101 10.1016/j.jss.2010.03.058 20605586 PMC4373617

[B9] Hepner DL Concepcion M Bhavani-Shankar K Coagulation status using thromboelastography in patients receiving warfarin prophylaxis and epidural analgesia J Clin Anesth 2002 14 6 405 410 10.1016/S0952-8180(02)00373-2 12393106

[B10] Wilson D Cooke EA McNally MA Wilson HK Yeates A Mollan RA Changes in coagulability as measured by thromboelastography following surgery for proximal femoral fracture Injury 2001 32 7650 10.1016/s0020-1383(01)00139-5 11754883

[B11] Muntz J Thromboprophylaxis in orthopedic surgery: how long is long enough? Am J Orthop (Belle Mead NJ) 2009 38 8 394 401 19809604

[B12] Martinelli I Bucciarelli P Mannucci PM Thrombotic risk factors: basic pathophysiology Crit Care Med 2010 38 2 Suppl S3 S9 10.1097/CCM.0b013e3181c9cbd9 20083911

[B13] Kashuk JL Moore EE Sabel A Barnett C Haenel J Le T Pezold M Lawrence J Biffl WL Cothren CC Johnson JL Rapid thrombelastography (r-TEG) identifies hypercoagulability and predicts thromboembolic events in surgical patients Surgery 2009 146 4 764 772 10.1016/j.surg.2009.06.054 19789037

[B14] Burden of illness in venous thromboembolism in critical care: a multicenter observational study J Crit Care 2005 20 341 347 10.1016/j.jcrc.2005.09.014 16310605

[B15] Park MS Martini WZ Dubick MA Salinas J Butenas S Kheirabadi BS Pusateri AE Vos JA Guymon CH Wolf SE Mann KG Holcomb JB Thromboelastography as a better indicator of postinjury hypercoagulable state than prothrombin time or activated partial thromboplastin time J Trauma 2009 67 2 266 276 10.1097/TA.0b013e3181ae6f1c 19667878 PMC3415284

[B16] Dai Y Lee A Critchley LA White PF Does thromboelastography predict postoperative thromboembolic events? A systematic review of the literature Anesth Analg 2009 108 3 734 742 10.1213/ane.0b013e31818f8907 19224777

[B17] Rafiq S Johansson PI Zacho M Stissing T Kofoed K Lilleør NB Steinbrüchel DA Thrombelastographic haemostatic status and antiplatelet therapy after coronary artery bypass surgery (TEG-CABG trial): assessing and monitoring the antithrombotic effect of clopidogrel and aspirin versus aspirin alone in hypercoagulable patients: study protocol for a randomized controlled trial Trials 2012 13 48 10.1186/1745-6215-13-48 22540524 PMC3502390

[B18] Lowe GDO Common risk factors for both arterial and venous thrombosis Br J Haematol 2008 140 488 495 10.1111/j.1365-2141.2007.06973.x 18275426

[B19] López JA Kearon C Lee AY Deep venous thrombosis Hematology Am Soc Hematol Educ Program 2004 2004 1 439 456 10.1182/asheducation-2004.1.439 15561697

[B20] Chirinos JA Heresi GA Velasquez H Jy W Jimenez JJ Ahn E Horstman LL Soriano AO Zambrano JP Ahn YS Elevation of endotheial microparticles, platelet, and leukocyte activation in patients with venous thromboembolism J Am Coll Cardiol 2005 45 9 1467 1471 10.1016/j.jacc.2004.12.075 15862420

[B21] Takahashi M Yamashita A Moriguchi-Goto S Marutsuka K Sato Y Yamamoto H Koshimoto C Asada Y Critical role of von Willebrand factor and platelet interaction in venous thromboembolism Histo Histopathol 2009 24 11 1391 1398 10.14670/HH-24.1391 19760588

[B22] Myers DD Jr Rectenwald JE Bedard PW Kaila N Shaw GD Schaub RG Farris DM Hawley AE Wrobleski SK Henke PK Wakefield TW Decreased venous thrombosis with an oral inhibitor of P selectin J Vasc Surg 2005 42 329 336 10.1016/j.jvs.2005.04.045 16102635

[B23] Bozic KJ Vail TP Pekow PS Maselli JH Lindenauer PK Auerbach AD Does aspirin have a role in venous thromboembolism prophylaxis in total knee arthroplasty patients? J Arthroplasty 2010 25 7 1053 1060 10.1016/j.arth.2009.06.021 19679434 PMC4142798

